# Genomic Epidemiology and Phenotyping Reveal on-Farm Persistence and Cold Adaptation of Raw Milk Outbreak-Associated *Yersinia pseudotuberculosis*

**DOI:** 10.3389/fmicb.2019.01049

**Published:** 2019-05-14

**Authors:** Hanna Castro, Anniina Jaakkonen, Anna Hakakorpi, Marjaana Hakkinen, Joana Isidro, Hannu Korkeala, Miia Lindström, Saija Hallanvuo

**Affiliations:** ^1^Department of Food Hygiene and Environmental Health, Faculty of Veterinary Medicine, University of Helsinki, Helsinki, Finland; ^2^Microbiology Unit, Laboratory and Research Division, Finnish Food Authority, Helsinki, Finland; ^3^National Reference Laboratory for Gastrointestinal Infections, Department of Infectious Diseases, National Institute of Health Dr. Ricardo Jorge, Lisbon, Portugal; ^4^Innovation and Technology Unit, Department of Human Genetics, National Institute of Health Dr. Ricardo Jorge, Lisbon, Portugal

**Keywords:** whole genome sequencing, phylogenomics, comparative genomics, stress tolerance, biofilm, growth modeling, food safety, raw milk

## Abstract

Packaged raw milk contaminated with *Yersinia pseudotuberculosis* mediated a large yersiniosis outbreak in southern Finland in 2014. The outbreak was traced back to a single dairy farm in southern Finland. Here we explore risk factors leading to the outbreak through epidemiologic investigation of the outbreak farm and through genomic and phenotypic characterization of the farm’s outbreak and non-outbreak associated *Y. pseudotuberculosis* strains. We show that the outbreak strain persisted on the farm throughout the 7-month study, whereas the non-outbreak strains occurred sporadically. Phylogenomic analysis illustrated that the outbreak strain was related to previously published genomes of wild animal isolates from Finland, implying that wild animals were a potential source of the outbreak strain to the farm. We observed allelic differences between the farm’s outbreak and non-outbreak strains in several genes associated with virulence, stress response and biofilm formation, and found that the outbreak strain formed biofilm *in vitro* and maintained better growth fitness during cold stress than the non-outbreak strains. Finally, we demonstrate the rapid growth of the outbreak strain in packaged raw milk during refrigerated storage. This study provides insight of the risk factors leading to the *Y. pseudotuberculosis* outbreak, highlights the importance of pest control to avoid the spread of pathogens from wild to domestic animals, and demonstrates that the cold chain is insufficient as the sole risk management strategy to control *Y. pseudotuberculosis* risk associated with raw drinking milk.

## Introduction

In spring 2014, Finland witnessed an outbreak of yersiniosis caused by *Yersinia pseudotuberculosis* and affecting 55 confirmed human cases (Pärn et al., 2015). The outbreak was quickly attributed to raw drinking milk packaged in 3-liter bag-in-boxes distributed in supermarkets across southern Finland. The raw milk packages were sourced back to a single Finnish dairy farm with 90 cows. Upon identification of the source, the producer voluntarily closed the raw milking packaging operation, and recalled packages already placed on the market. Sampling of the farm revealed that the farm’s bulk tank milk was contaminated with the outbreak strain at the level of 2 CFU/ml, and that *Y. pseudotuberculosis* was also present in the milk filters and in cow feces (Pärn et al., 2015).

*Yersinia pseudotuberculosis* is a gram-negative zoonotic foodborne pathogen and the predecessor of *Y. pestis*, the causative agent of bubonic plague (Achtman et al., 1999). In the human host, *Y. pseudotuberculosis* causes gastrointestinal symptoms that are frequently misdiagnosed as appendicitis, resulting in unnecessary appendectomies (Nuorti et al., 2004). Complications such as reactive arthritis or erythema nodosum occasionally follow the gastrointestinal symptoms (Tertti et al., 1984; Jalava et al., 2006). *Y. pseudotuberculosis* can survive and proliferate outside the host, form biofilms, and develop resilience against a variety of environmental stressors (Joshua et al., 2015; Chen et al., 2016; Zhao et al., 2017). The psychrotrophic nature of *Y. pseudotuberculosis* presents a challenge for the control the pathogen in food production (Palonen et al., 2010; Keto-Timonen et al., 2016).

*Yersinia pseudotuberculosis* can infect the majority of domestic animal species, including cattle (Hodges et al., 1984; Tertti et al., 1984; Tsubokura et al., 1989). Wild animals play a key role in the ecology of *Y. pseudotuberculosis*, and a wide range of wild mammalian and avian species carry and disseminate the pathogen (Mair, 1973; Fukushima and Gomyoda, 1991; Fredriksson-Ahomaa et al., 2009). Indeed, exposure to wild animals is a risk factor for the occurrence of *Y. pseudotuberculosis* in domestic pigs (Laukkanen et al., 2008). *Y. pseudotuberculosis* is ubiquitous in the soil and water in natural environments and agroecosystems (Bercovier et al., 1978; Fukushima et al., 1995; Jalava et al., 2006). In Finland, the majority of *Y. pseudotuberculosis* outbreaks are linked to fresh produce (Jalava et al., 2004, 2006; Nuorti et al., 2004; Kangas et al., 2008). The aforementioned outbreak of spring 2014 was the first *Y. pseudotuberculosis* outbreak linked to packaged raw milk in Finland, and presumably world-wide. *Y. pseudotuberculosis* is an uncommon contaminant of raw milk (Greenwood and Hooper, 1989; Hamama et al., 1992; Ruusunen et al., 2013; Jamali et al., 2015; Bonardi et al., 2018), although it is occasionally detected in bovine feces (Hodges and Carman, 1985; Callinan et al., 1988). Consequently, the ecology and transmission routes of *Y. pseudotuberculosis* on dairy farms remain widely uninvestigated.

In the present study, we use epidemiological investigation, phenotyping, and whole genome sequencing to investigate the epidemiology and characteristics of *Y. pseudotuberculosis* on the source farm of the spring 2014 outbreak. We found that the outbreak strain persisted on the source farm through the entire 7-month study, whereas other *Y. pseudotuberculosis* strains on the farm occurred only sporadically. Phylogenomic analysis illustrated that the outbreak strain was related to previously published genomes of wild animal isolates from Finland, implying wild animals as a potential source of the outbreak strain to the farm. Comparative genomics revealed that several genes associated with virulence, stress response and biofilm formation differed between the outbreak, and non-outbreak associated strains from the investigated farm. Phenotypic screening provided evidence of strain characteristics favorable for the persistence of the outbreak strain on the source farm, namely the ability to form biofilm *in vitro* and increased growth fitness at low temperature. Finally, we explored the role of consumer storage in the outbreak by demonstrating the rapid growth of the outbreak strain in packaged raw milk during refrigerated storage.

## Materials and Methods

### Follow-Up Study of the Outbreak Source Farm

A total of 234 samples were collected in a follow-up investigation of the outbreak dairy farm and raw milk packaging facility between April 7, 2014 and November 17, 2014 (Table 1). Collection and microbiological analysis of the samples collected on April 7 from raw milk (*n* = 8), milk filters (*n* = 3), and cow feces (*n* = 9) were published previously (Pärn et al., 2015). Molecular typing with pulsed-field gel electrophoresis (PFGE) indicated that the fingerprint profile (i.e., the pulsotype) of the milk and milk filter isolates collected on April 7 was identical to that of the outbreak-associated human isolates (Pärn et al., 2015).

**Table 1:** Detection of *Yersinia pseudotuberculosis* in samples collected from the outbreak source farm and raw milk packaging facility during April – November, 2014 (number of positive samples/number of samples collected, *N* = 234).

Sample type	Sampling date								
	7.4	17.4	23.4	29.4	26.5	16.6	9.10	21.10	17.11
Raw milk samples									
Packaged milk	3/3^a,b^	1/1^c^		1/1^d^					
Bulk tank milk	5/5^a^		5/5		3/3	0/5		5/5	0/5
Individual cows			0/10						
Milk filter socks	3/3^a^		2/2		2/3	0/2	3/3	3/3	2/2
Fecal samples	2/9^a^				0/90	1/6	0/3		
Milking system	0/8		0/6			0/9			
Barn environment	0/2		0/10		0/4	0/15			
Packaging facility						0/11			

^a^*Data published by Pärn et al. (2015).*^b^*Packages obtained from the producer’s storage; packaging date March 31, 2014*. ^c^*Package obtained from a consumer’s refrigerator; packaging date March 24, 2014.*^d^*Package obtained from a consumer’s refrigerator; packaging date March 27, 2014*.

The methodology employed by Pärn et al. (2015) for the isolation and identification of *Y. pseudotuberculosis* was used for the remaining 214 samples. In brief, the samples were homogenized in phosphate-mannitol-peptone broth and plated directly on cefsulodin irgasan novobiocin (CIN) agar (Oxoid, United Kingdom). The broth was cold-enriched at 4°C for 7 and 14 days. Alkali-treated samples (0.5 ml of the broth was mixed with 4.5 ml of 0.25% KOH solution for 20 s) were streaked onto CIN agar and incubated at 30°C for 48 h. Five aliquots of milk were analyzed from each raw milk package. Subsets of 10-g fecal samples, 25-ml milk and water samples, milk filters, and environmental swabs were enriched. *Y. pseudotuberculosis* were identified using API 20E (bioMerieux, France) and MALDI-Biotyper (Bruker Daltonics GmbH, Switzerland). For each positive sample, a minimum of three isolates of *Y. pseudotuberculosis* were subtyped with PFGE using restriction enzymes SpeI and NotI, as described by Fredriksson-Ahomaa et al. (1999). BioNumerics 6.6 (Applied Maths, Belgium) was used for fingerprint analysis, where pulsotypes were discerned from one another at the level of single band differences.

*Yersinia pseudotuberculosis* were enumerated using average plate counts on duplicate CIN agar plates. Additionally, the most probable number (MPN) of *Y. pseudotuberculosis* in milk were determined using a MPN calculator (Jarvis et al., 2010), from triplicate tubes containing 1 ml of milk and 9 ml of phosphate-mannitol-peptone (FMP) solution that were incubated in 4°C for 14 days. Standard methods were employed to enumerate total bacterial (International Organisation for Standardization [ISO], 2003) and enterobacteria (International Organisation for Standardization [ISO], 2004) in milk.

Farm staff were interviewed about the hygienic maintenance of the automated milking system employed by the farm. The milking system was washed automatically every 8 h with a solution containing 5–10% sodium hydroxide and 2.5–5% sodium hypochlorite. For every third wash, the alkaline detergent was replaced by a solution containing 5–20% nitric acid, 5–10% sulfuric acid, and <10% phosphoric acid. The milking system was serviced by the manufacturer every 6 months. Milk filters were replaced thrice daily. The farm collected milk into a single bulk tank, which was replaced with a new tank in April 2014.

### Whole Genome Sequencing

Ten farm isolates with the outbreak pulsotype (Pärn et al., 2015) from packaged raw milk (*n* = 3), bulk tank milk (*n* = 3) and milk filters (*n* = 4), and four isolates with non-outbreak associated pulsotypes from feces, were selected for genome analysis using whole genome sequencing (Table 2). DNA was extracted using the PureLink Genomic DNA Mini Kit (Thermo Fisher Scientific, United States) according to manufacturer’s instructions. Genomic libraries were prepared from the DNA samples using the Nextera XT DNA Sample Preparation Kit (Illumina, United States), and paired-end sequencing (2 bp × 250 bp) was performed using the Illumina MiSeq platform.

**Table 2:** *Yersinia pseudotuberculosis* isolates from the outbreak farm selected for analysis using whole genome sequencing (*N* = 14).

ID	Sampling date	Source	PFGE^a^	MLST^b^	Serotype	pYV^c^
S1	7.4.2014	Bulk tank milk	S-1/N-1^d^	43	O:1b	Present
S2	7.4.2014	Milk filter	S-1/N-1	43	O:1b	Present
S4	17.4.2014	Milk package	S-1/N-1	43	O:1b	Present
S7	7.4.2014	Milk package	S-1/N-1	43	O:1b	Present
S8	23.4.2014	Bulk tank milk	S-1/N-1	43	O:1b	Present
S9	23.4.2014	Milk filter	S-1/N-1	43	O:1b	Present
S10	29.4.2014	Milk package	S-1/N-1	43	O:1b	Present
S13	26.5.2014	Milk filter	S-1/N-1	43	O:1b	Absent
S18	21.10.2014	Bulk tank milk	S-1/N-1	43	O:1b	Absent
S23	17.11.2014	Milk filter	S-1/N-1	43	O:1b	Absent
S24	7.4.2014	Feces	S-2/N-2	42	O:1a	Present
S25	7.4.2014	Feces	S-2/N-2	42	O:1a	Present
S26	7.4.2014	Feces	S-2/N-2	42	O:1a	Present
S27	16.6.2014	Feces	S-3/N-3	42	O:1a	Present
IP32953		Human		42	O:1b	Present

*MLST and serotype were determined *in silico* from the assemblies. Reference genome of the strain IP32953 was included in the analysis*. ^a^*PFGE, pulsed-field gel electrophoresis.*^c^*MLST, multi-locus sequence typing.*^c^*pYV, *Yersinia pseudotuberculosis* pYV plasmid sequence (NC_006153.2).*^d^*(S-1/N-1), outbreak genotype (Pärn et al., 2015)*.

The Illumina primary QA/QC analysis was used as initial quality control of the raw reads. Reads that passed the initial quality control were then exposed to quality control and *de novo* assembly using the INNUca QA/QC 3.1 pipeline (Machado et al., 2017). In brief, the pipeline involved adapter removal and trimming of low quality reads using Trimmomatic 0.36 (Bolger et al., 2014), assembly using SPAdes 3.9 (Bankevich et al., 2012), and assembly correction using Pilon 1.18 (Walker et al., 2014). The assembled draft genomes were annotated using Prokka 1.12 (Seemann, 2014), and assembly quality metrics were obtained using QUAST 4.0 (Gurevich et al., 2013; Supplementary Table S1) 2.3 *in silico* subtyping.

Multi-locus sequence typing (MLST) analysis of the draft genomes was done using an established schema (Laukkanen-Ninios et al., 2011) in the Enterobase platform (Alikhan et al., 2018). Serotyping was done *in silico* by aligning the draft assemblies against *Y. pseudotuberculosis* O-antigen sequence clusters (Kenyon et al., 2017) using BLASTn (Boratyn et al., 2013).

### Phylogenomic Analyses

The Lyve-set 1.1.4f pipeline (Katz et al., 2017) was used to infer the phylogenies of ST42 and ST43 isolates. Phylogenies of the two sequence types were constructed separately. IP32953 (NZ_CP009712; Johnson et al., 2015) was used as the reference genome in the phylogenomics analyses of ST42 isolates. Since no complete genome was available for ST43, a high-quality draft genome assembly (YER_CA8000AA_AS) obtained from the Enterobase database (Alikhan et al., 2018) was used as the ST43 reference genome. Input for the analysis consisted of fastq-files of the 14 sequenced isolates from the present study, and 55 previously sequenced *Y. pseudotuberculosis* isolates of ST42 (*n* = 29) and ST43 (*n* = 26) isolates (Seecharran et al., 2017; Williamson et al., 2017; Supplementary Tables S2, S3). The Lyve-set 1.1.4f pipeline (Katz et al., 2017) was run using default settings with options mask-phages, mask-cliffs, and read_cleaner CGP. The pipeline detected high-quality single-nucleotide polymorphisms (hqSNPs), calculated pairwise distances (PWD), and constructed a Maximum Likelihood tree using RAxML 8.1.16 (Stamatakis, 2014) using the GTR-model with 500 bootstrap replicates. Trees were visualized using FigTree 1.4.3 (Rambaut, 2014).

### Genomic Characterization

Core genes of 14 *Y. pseudotuberculosis* isolates from the outbreak farm (Table 2) and the reference genome IP32953 were aligned using Roary 3.8.0 (Page et al., 2015). Roary was executed with option -e and -z to generate a core gene alignment file and alignment files for individual genes using PRANK (Löytynoja, 2014). Variant sites within 110 genes associated with virulence, stress response and biofilm formation (Supplementary Table S4) were identified from the alignment files using MEGA 7 (Kumar et al., 2016). The list of 110 genes was gathered through a literature review conducted using Google Scholar with key words “*Y. pseudotuberculosis*,” “virulence,” “Stress,” and “Biofilm.” Translated nucleotide sequence identities (BLASTx identity) between alignments were determined using BLASTx (Boratyn et al., 2013). Isolates were screened for the presence of the pYV plasmid (NC_006153.2) by aligning contigs against the plasmid using BLASTn. The presence/absence of the pYV plasmid was confirmed experimentally using the CR-MOX test (Riley and Toma, 1989).

### Biofilm Formation

*In vitro* biofilm formation by the 14 sequenced *Y. pseudotuberculosis* isolates (Table 2) was investigated using a microtiter plate assay, as described by Hinchliffe et al. (2008), with modifications. In brief, six biological replicates of each isolate were pre-incubated in LB broth (Sigma-Aldrich, Germany) at 30°C for 22 h. The cultures were diluted in LB broth and 75 μl of the dilution was transferred onto 96-well polystyrene microtiter plates (Corning 3598; Sigma-Aldrich). The reference strain IP32953, known to produce robust biofilms *in vitro* (Hinchliffe et al., 2008), was included on each microtiter plate as a positive control. Additionally, each plate contained uninoculated LB broth as a negative control. The plates were incubated at 28°C for 24 h, after which the plates were washed with buffered peptone solution (BPS), dried for 20 min and stained with 0.4% crystal violet for 15 min. Following three more washes with BPS, the stained biofilm was suspended in 100 μl of a solution containing 10% acetic acid and 30% methanol, and the OD_595nm_ was measured using a spectrophotometer (Multiskan EX, Thermo Fisher Scientific). To minimize the effect of background interference, the absorbance levels of the negative controls were subtracted from the test wells prior to data analyses.

### Growth at 3°C

Twenty-nine isolates from the follow-up investigation, representing different sampling dates and sources (Supplementary Table S5), were selected for the growth study at 3°C using Bioscreen C MBR (Growth Curves Ltd, Finland). The ST42 *Y. pseudotuberculosis* reference strain IP32953 was used as a positive control. We previously demonstrated that colony counts of IP32953 grown at 3°C correspond with the OD_600nm_ values obtained by Bioscreen MBR (Palonen et al., 2011). In the present study, preparation of the cultures and the growth study at 3°C were performed as described previously (Palonen et al., 2011). In brief, three biological replicates of each strain were grown in LB broth for 16 h at 28°C, diluted in LB broth to an initial OD_600nm_ value of 0.017, and pipetted onto microtiter plates in 300-μl portions. Replicates were spread across the microtiter plates to avoid location bias. Microtiter plates were incubated for 21 days at 3°C in the Bioscreen C MBR, which measured the turbidity within the microtiter wells hourly at 600 nm. Uninoculated LB broth was used as a negative control.

### Growth of the Outbreak Strain in Packaged Raw Milk

Growth of the outbreak strain isolate S4 (Table 2) was investigated in refrigerated raw milk packaged in 1-liter plastic bottles and 3-liter bag-in-boxes. The isolate was inoculated into separate raw milk bottles and bag-in-boxes to targeted inoculum levels of 0.3, 1.3, and 2.3 log CFU/ml. The growth study was triplicated at each inoculum level so that in total nine bottles and nine bag-in-boxes were inoculated. To optimize freshness, the growth study was initiated on the same day the milk packages were delivered from the dairy to the retail store from which the packages were purchased. Control samples were taken from each package to confirm that the milk initially was free of *Yersinia*. Control samples were analyzed as previously described (Pärn et al., 2015). The outbreak strain was cultured in BHI at 30°C for 24 h to the level of 7 log CFU/ml and then diluted in isotonic saline to the appropriate target levels. Bag-in-boxes were inoculated with a sterile needle and syringe, and bottles were inoculated by pipetting. The packages were stored at 6°C and sampled on days 0, 3, 5, and 7 to determine *Y. pseudotuberculosis* growth. Upon sampling, 10 ml of milk was collected from the bottles and 30 ml were collected from the bag-in-boxes. Colony counts of *Y. pseudotuberculosis* were determined using a dilution series with duplicate plating on CIN-agar. The pH of each milk sample was measured using the inoLab^®^ pH 7110 (Xylem Analytics, United States) pH meter, which was calibrated daily using technical buffers (Xylem Analytics). The growth of aerobic bacteria in uninoculated raw milk bottles and bag-in-boxes from the same producer were investigated previously (Castro et al., 2017).

### Data Analyses

Absorbance data obtained from Bioscreen C MBR were analyzed using the Grofit package (Kahm et al., 2010) in R 3.3.2 (R Core Team, 2018). Growth parameters, namely maximum growth rate, lag time, asymptote, and area under curve (AUC) were determined for each biological replicate using spline values. For growth studies in packaged raw milk, standard deviations were calculated from log-transformed colony count data. If no colonies were detected in a given sample, -0.3 log CFU/ml was used as the log-transformed value for the calculation. The maximum growth rates in packaged raw milk were determined by fitting the Baranyi and Roberts model (Baranyi and Roberts, 1994) into the colony count data using the Combase DMFit software^1^. Statistical analyses of data were run on the SPSS Statistics 24 software (IBM, NY). Normalization of data were evaluated using Kolmogorov-Smirnov and Shapiro-Wilkin tests of normalization.

## Results

### Epidemiological Investigation in the Outbreak Farm

*Yersinia pseudotuberculosis* was detected in 41 (18%) of the 234 samples collected during the follow-up study (Table 1). *Y. pseudotuberculosis* was detected in samples of milk, milk filters, and bovine feces, whereas all environmental swab samples from the farm environment or packaging facility were negative for *Y. pseudotuberculosis*. PFGE pulsotyping with restriction enzymes *SpeI* and *NotI* revealed that the outbreak pulsotype S-1/N-1 occurred frequently in milk and milk filter samples throughout the 7-month study but was not detected in bovine feces (Supplementary Figures S1, S2). In contrast, the two pulsotypes that were detected in bovine feces (S-2/N-2 and S-3/N-3) occurred sporadically and were not isolated from the milk or milk filters. Pulsotype S-2/N-2 was detected in the feces of lactating cows, while pulsotype S-3/N-3 occurred in calf feces.

The low levels of total bacteria (5700 CFU/ml) and enterobacteria (50 CFU/ml) recovered from bulk tank milk on April 7th were not suggestive of poor milking hygiene despite the presence of *Y. pseudotuberculosis*. The levels of *Y. pseudotuberculosis* in bulk tank milk and milk filter samples decrease during the course of the study. On April 7th, *Y. pseudotuberculosis* levels were 2 CFU/ml in bulk tank milk (Pärn et al., 2015) and 10^5^ CFU/filter in milk filters. In October, the levels of *Y. pseudotuberculosis* were 0.4 CFU/ml in bulk tank milk, and between 10^2^ and 10^4^ in milk filters. At the end of the study in November, *Y. pseudotuberculosis* was no longer recovered from milk and levels in milk filters were below 20 CFU/filter. Packaged raw milk (packaging date March 24th) obtained from a consumer’s refrigerator on April 17th contained *Y. pseudotuberculosis* levels of 10^6^ CFU/ml. *Y. pseudotuberculosis* were not enumerated from other raw milk packages.

### *In silico* Subtyping and Phylogenomics

**Figure 1:**
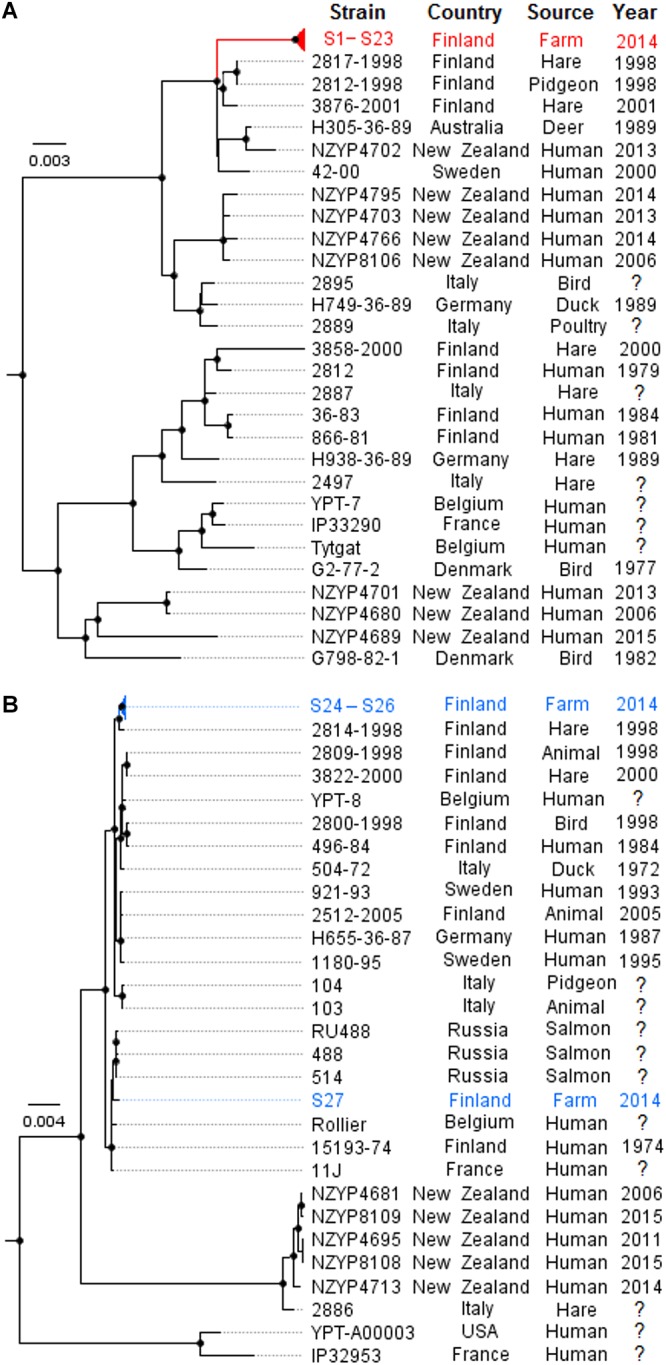
Phylogenomic analysis of 14 outbreak farm isolates and previously published *Yersinia pseudotuberculosis* ST43 (*n* = 29) and ST42 (*n* = 27) genomes by maximum likelihood. Separate phylogenomic analyses were made for ST43 **(A)** and ST42 **(B)**. Phylogenies were inferred using SNP alignment files generated by the Lyve-SET 1.1.4f pipeline, using RAxML 8.1.16 with 500 bootstrap replicates, and the trees were visualized using FigTree 1.4.3. Convergence was reached after 400 bootstrap replicates for ST43 **(A)** and 100 bootstrap replicates for ST42 **(B)**. The tree is drawn to scale and rooted to midpoint; nodes labeled with black circles indicate bootstrap values above 80%. Outbreak farm isolates from milk and milk filters are demarcated red; fecal isolates from the outbreak farm are demarcated blue. Phylogenomics of the ST43 strains confirmed that the milk and milk filter isolates S1–S23 from the outbreak farm formed a single outbreak cluster **(A)**. Conversely, ST42 fecal isolates S24–S26 from the outbreak farm did not share a common recent ancestor with fecal isolate S27 **(B)**.

A subset of 14 isolates from the outbreak source farm were selected for whole genome sequencing (Table 2). *In silico* MLST and serotyping of the 14 isolates revealed that *Y. pseudotuberculosis* isolates of the outbreak pulsotype S-1/N-1 belonged to ST43 and contained the O-antigen gene cluster of serotype O:1b. In contrast, isolates of the pulsotypes S-2/N-2 and S-3/N-3 belonged to ST42 and contained the O-antigen gene cluster of serotype O:1a. Phylogenomic analyses demonstrated that isolates having the outbreak pulsotype S-1/N-1 clustered into a single subclade (the “outbreak cluster”), in which PWDs between isolates were 0-3 SNPs (Figure 1). In contrast, PWDs between the outbreak cluster and other ST43 genomes ranged from 46 to 350 SNPs (mean 215 SNPs). Three wild animal isolates from Finland bore the greatest resemblance to the outbreak cluster (PWD 46–70 SNPs). However, the distance to the outbreak cluster was within a similar range for other European and Oceanic isolates of the same clade (PWDs 58–98 SNPs). The closest relative of the ST42 fecal isolates S24–S26 was a Finnish wild hare isolate (PWDs 66–71 SNPs), while the fecal isolate S27 bore the closest resemblance to a Finnish human isolate (PWD 45 SNPs).

### Genomic Characterization

The 14 sequenced isolates from the outbreak farm were screened for the presence and diversity of the *Y. pseudotuberculosis* derived mitogen (YMP) and 110 other genes associated with virulence, stress response, or biofilm production. Whereas the YMP genes were not detected in any isolate, both the ST43 outbreak cluster and the ST42 fecal isolates contained the *Yersinia* high pathogenicity island (HPI) and the virulence genes associated with the plasmid pYV. Interestingly, the outbreak cluster isolates from May 26th onward (S13, S18, and S23) lacked the pYV-associated genes. Results of the CR-MOX test confirmed the absence of the plasmid in these isolates, indicating that loss of pYV occurred either on the farm or during sample processing. Aside from the loss of this plasmid, the 110 genes associated with virulence, stress tolerance and biofilm formation were identical among isolates of the outbreak cluster. Allelic diversity between the ST43 outbreak cluster and the IP32953 reference genome was limited to ≤9 SNPs in 34/110 genes (Supplementary Table S4), which translated to ≤6 non-synonymous mutations in a total of 14 genes (Table 3). In contrast, the ST42 fecal isolates and the reference genome contained marked allelic diversity in genes encoding a two-partner secretion system for *Serratia*-like hemolysin: 48 non-synonymous mutations were detected in *shlB*_1_, 10 in *shlA_1_*_,_ and 11 in *shlA_2_*. Moreover, nine non-synonymous mutations between the ST42 fecal isolates and IP325953 were present in the *lcrV* virulence gene encoding a pYV-associated V-antigen. Overall, 8 genes contained non-synonymous mutations between the reference genome and the fecal isolates (≤128 SNPs in 14/110 genes).

**Table 3:** Non-synonymous mutations between IP32953 and outbreak farm isolates belonging to ST42 (*n* = 4) and ST43 (*n* = 10) among 110 genes associated with virulence, stress response, biofilm formation, and motility.

Gene name	Refseq locus tag	Functional role			NS sites^a^		References
		Virulence	Stress response	Biofilm/motility	ST42	ST43	
*ascD*	YPTB_RS05510			✓	0	1	Joshua et al., 2015
*cckA*	YPTB_RS20595			✓	1	0	Joshua et al., 2015
*cheA*	YPTB_RS13085	✓	✓		0	5	Palonen et al., 2011
*clsA*	YPTB_RS11560	✓			0	2	Revel and Miller, 2001
*clpV*	YPTB_RS03625	✓	✓		0	1	Chen et al., 2016
*hmsF*	YPTB_RS10700			✓	0	1	Zhao et al., 2017
*hmsP*	YPTB_RS20735			✓	5	6	Zhao et al., 2017
*hmsS*	YPTB_RS10710			✓	0	1	Zhao et al., 2017
*lcrV*	YPTB_RS21700	✓(pYV)^b^		✓	9	0	Atkinson and Williams, 2016
*nhaB*	YPTB_RS11345		✓		0	6	Chen et al., 2016
*rstB*	YPTB_RS12170		✓		3	0	Chen et al., 2016
*shlA1*	YPTB_RS11125	✓(?)^c^			10	0	Di Venanzio et al., 2014
*shlA2*	YPTB_RS19745	✓(?)			11	0	Di Venanzio et al., 2014
*shlB1*	YPTB_RS11120	✓(?)			48	0	Di Venanzio et al., 2014
*shlB2*	YPTB_RS19740	✓(?)			1	1	Di Venanzio et al., 2014
*topA*	YPTB_RS11700	✓			0	1	Revel and Miller, 2001
*yadA*	YPTB_RS21525	✓(pYV)		✓	0	1	Atkinson and Williams, 2016
*ybtP*	YPTB_RS08795	✓ (HPI)^d^			0	1	Revel and Miller, 2001
*yscP*	YPTB_RS21755	✓(pYV)			0	1	Atkinson and Williams, 2016
*yscQ*	YPTB_RS21760	✓(pYV)			0	3	Atkinson and Williams, 2016

^a^*NS sites, number of sites containing non-synonymous mutations between the Refseq sequence and the ST42/ST32 farm isolates*. ^b^*(pYV), virulence gene associated with plasmid pYV*. ^c^*(?), virulence gene characterized in *Serratia*; role in *Y. pseudotuberculosis* unknown*. ^d^*(HPI), virulence gene associated with the *Y. pseudotuberculosis* high pathogenicity island*.

### *In vitro* Biofilm Formation

**Figure 2:**
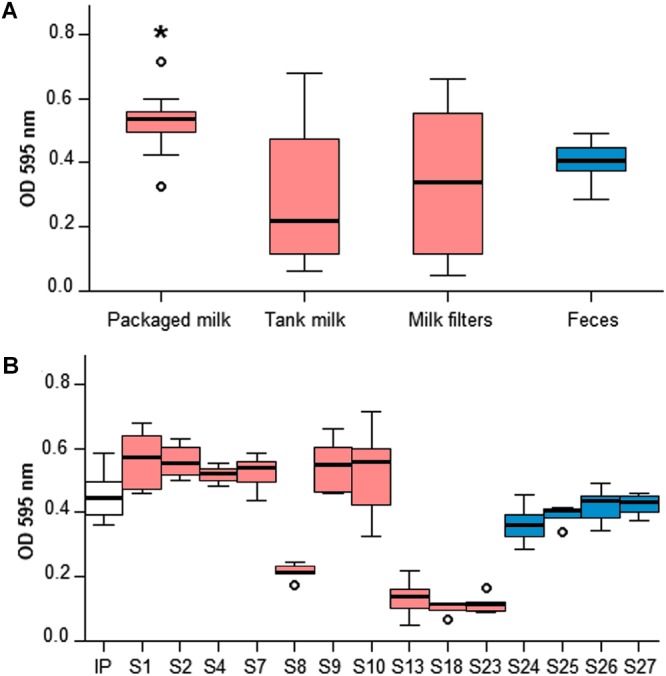
Crystal violet microtiter plate assay of 14 *Y. pseudotuberculosis* isolates from the outbreak source farm. *In vitro* biofilm formation was investigated on 96-well microtiter plates cultured at 30°C for 24 h. Absorbance (OD_595nm_) levels were measured using an automatic plate reader following staining in 0.4% crystal violet and dilution in a solution of 10% acetic acid and 30% methanol. Six biological replicates of each isolate were included and IP32952 (IP) was used as a positive control. Box plots colors indicate isolate source: red for milk and milk filters; blue for feces. The asterisk (^∗^) indicates statistical siginificance (Kruskal-Wallis Test, *p* < 0.05). Absorbance levels were significantly higher for ST43 isolates from packaged raw milk than ST42 isolates from feces **(A)**. ST43 isolates from bulk tank milk and milk filters separated into two distinct phenotypes producing high (S1, S2, and S9) and low (S8, S13, S18, and S23) absorbance levels **(B)**.

Crystal violet microtiter plate assay demonstrated that different *in vitro* biofilm forming phenotypes were present among the 14 *Y. pseudotuberculosis* farm isolates included in the analysis (Figure 2). Packaged raw milk isolates, all pYV(+), produced significantly higher absorbance levels than the pYV(+) isolates from feces (Independent-samples Kruskal-Wallis Test, *p* < 0.05). The bulk tank milk and milk filter isolates of the outbreak cluster separated into good and poor *in vitro* biofilm formers, where isolates that lacked the virulence plasmid pYV (S13, S18, S23) had the poorest biofilm forming ability. Curiously, the pYV(+) isolate S8 had a similar phenotype to the pYV(-) isolates. The possibility that S8 lost the plasmid during sample preparation for the assay was not excluded.

### Growth Characteristics at 3°C

**Figure 3:**
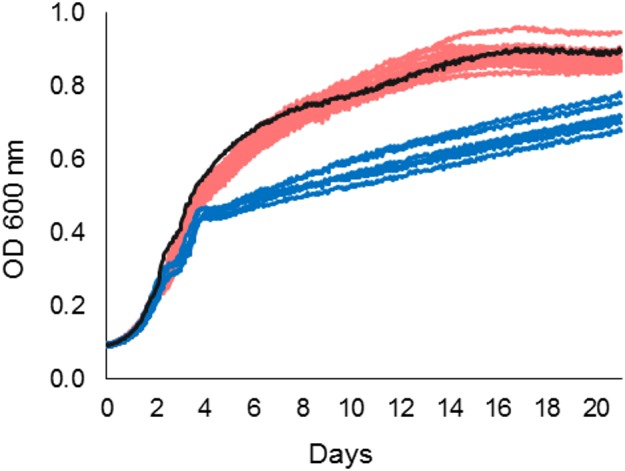
Growth of 29 *Y. pseudotuberculosis* isolates from the outbreak source farm at 3°C. Growth data was obtained by Bioscreen C MBR as measurements of optical density (OD) at 600 nm. ST43 isolates from milk and milk filters (S1–S23, red), reached higher absorbance levels than ST42 isolates from bovine feces (S24–S29, blue). *Y. pseudotuberculosis* reference strain IP32953 (black) acted as a positive control.

Growth characteristics of the outbreak farm isolates from milk (*n* = 11), milk filters (*n* = 12), and feces (*n* = 6) were investigated at 3°C. The shape of the growth curves differed between the ST43 outbreak cluster isolates and the ST42 fecal isolates (Figure 3). Whereas the ST43 isolates maintained exponential growth until asymptote, the growth of the ST42 isolates plateaued periodically on days 3 and 4. Consequently, milk and milk filter isolates reached significantly higher maximum growth rate, level of maximum growth (asymptote), and AUC, than isolates from feces (Mann-Whitney *U* Test, *p* < 0.01), suggesting that the ST43 outbreak strain had better growth fitness at 3°C than the ST42 fecal isolates.

### Growth in Packaged Raw Milk

**Figure 4:**
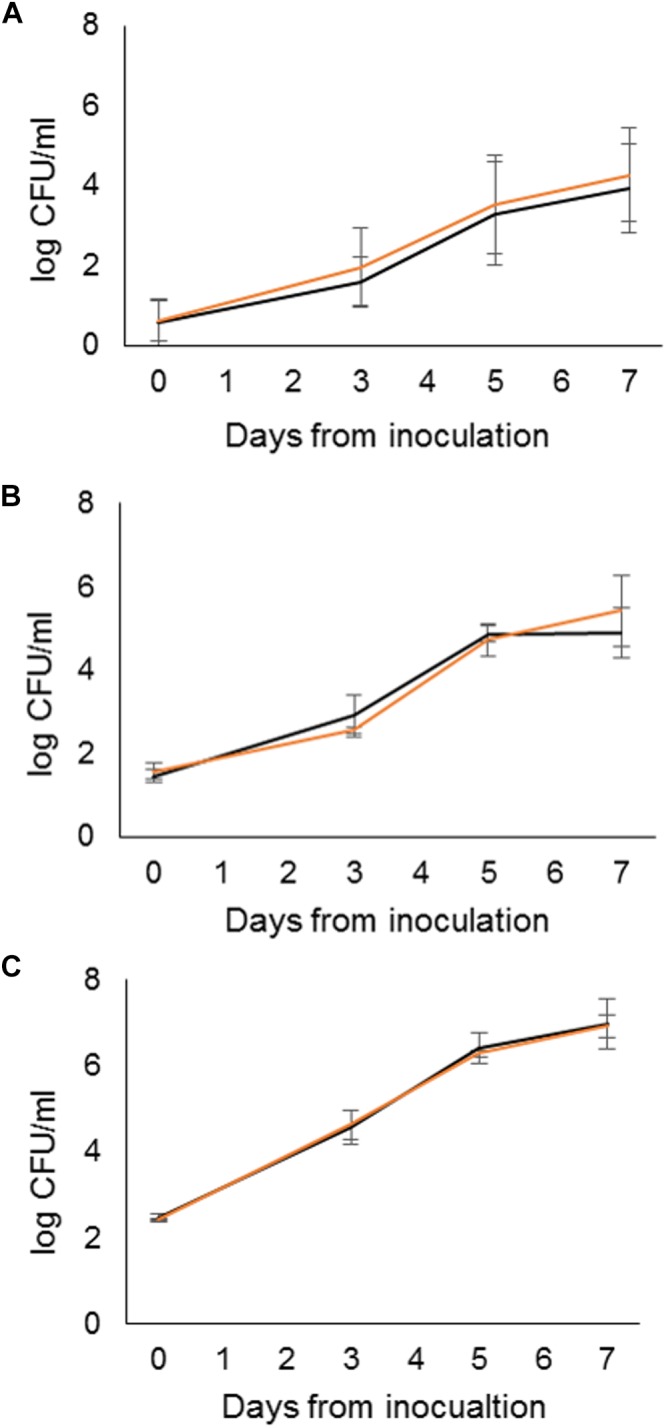
Growth characteristics of the ST43 packaged-raw-milk isolate S4 in raw milk bottles (black) and bag-in-boxes (orange) inoculated to target levels of 2 CFU/ml **(A)**, 20 CFU/ml **(B)**, and 200 CFU/ml **(C)** and stored at 6°C for 7 days, with sampling and enumeration of *Y. pseudotuberculosis* performed on days 0, 3, 5, and 7.

The growth of the outbreak strain in raw milk bottles and in bag-in-boxes stored at 6°C was investigated using the packaged raw milk isolate S4 (Table 2). S4 was inoculated into the packages at three targeted inoculum levels: 2, 20, and 200 CFU/ml. No statistically significant differences were observed between package types at any inoculum level. At the 2 CFU/ml target inoculum level (0.3 log CFU/ml), S4 grew during 7 days of storage from mean initial counts of 0.6 log CFU/ml (bottles) and 0.6 log CFU/ml (bag-in-boxes) to counts of 3.9 log CFU/ml (bottles), and 4.0 log CFU/ml (bag-in-boxes) (Figure 4). The maximum growth rate of S4 was 0.6 log CFU/ml/day in both package types. At the targeted inoculum level of 20 CFU/ml (1.3 log CFU/ml), S4 grew from mean initial counts of 1.5 log CFU/ml (bottles) and 1.6 log CFU/ml (bag-in-boxes) to mean counts of 4.9 log CFU/ml (bottles) and 5.4 log CFU/ml (bag-in-boxes) in 7 days. The maximum growth rate was 0.7 log CFU/ml/day in both package types. At the targeted inoculum level of 200 CFU/ml (2.3 log CFU/ml), S4 grew from mean initial counts of 2.5 log CFU/ml (bottles) and 2.4 log CFU/ml (bag-in-boxes) to mean counts of 7.0 log CFU/ml (bottles) and 6.9 log CFU/ml (bag-in-boxes) in 7 days. In both package types, S4 had a maximum growth rate of 0.8 log CFU/ml/day. The pH of milk remained nearly unchanged during the storage period: pH levels were 6.7–6.8 on day 0, and 6.6–6.7 on day 7.

## Discussion

The outbreak strain persisted on the source farm until the end of the study in November 17, 2014, 9 months after the first outbreak patient fell ill in February 2014 (Pärn et al., 2015). Since bulk tank milk is not routinely screened for the presence of *Y. pseudotuberculosis* (Pärn et al., 2015), milk contamination may have occurred even prior to the beginning of the raw milk sales operation. The low level genomic diversity within the outbreak cluster suggests that transmission to the farm occurred recently from a point source of contamination. Curiously, the outbreak strain was not identified in samples of bovine feces, the farm environment nor the packaging facility. The presence of other, non-outbreak-related strains in bovine feces indicates that the cattle were nevertheless exposed to *Y. pseudotuberculosis*. Hygienic measures were therefore insufficient to prevent the contamination of feeds or feeding areas. If shedding of the outbreak strain in feces occurred intermittently, the sampling frequency may have been insufficient to identify the outbreak strain in bovine feces.

Carriage of *Y. pseudotuberculosis* has previously been documented in barn rat populations (Kaneko et al., 1979). Upon sampling of the farm premises, we noted that feed storages were unprotected from wild birds and rodents, and rats were occasionally seen in the barn. From these observations we hypothesized that wild animals were a probable source of *Y. pseudotuberculosis* to the farm. Indeed, out of 27 previously published ST43 genomes, Finnish wild animal isolates bore the closest familiarity to the outbreak cluster, and although the degree of familiarity did not provide sufficient evidence for a strong epidemiologic link. The analysis was limited by the scarcity of publicly available *Y. pseudotuberculosis* genomes. Increasing the availability of sequencing data of *Y. pseudotuberculosis* strains from various hosts and locations would provide a baseline of strain diversity and facilitate source attribution in future outbreak investigation. Although the source of the outbreak could not be proven, the deficiencies in pest control, and feed hygiene indicate that the farm was at a high risk for the spread of pathogens from wild life to cattle.

*Yersinia pseudotuberculosis* mastitis in dairy cows is an exceedingly rare source of milk contamination (Messerli, 1972; Bleul et al., 2002; Sampimon et al., 2005; Shwimmer, 2007). In the present study, the recurrence of the outbreak strain in milk over 7 months dictates that mastitis was not a plausible source. Additionally, no evidence for *Y. pseudotuberculosis* mastitis were found in quarter milk samples, albeit the sampling was limited to ten cows (11% of the herd).

Both the ST43 outbreak cluster and the ST42 fecal isolates contained the complete HPI and, with the exception of three outbreak cluster isolates, the virulence plasmid pYV, suggesting that both the outbreak, and non-outbreak strains were highly pathogenic. In total, 19 genes associated with virulence, stress response and biofilm formation, contained non-synonymous mutations between the ST42 and ST43 farm isolates, which potentially contributed to the phenotypical, and epidemiological differences between the two groups. Of note, numerous non-synonymous mutations between the ST43 outbreak cluster and the ST42 fecal isolates were observed in the *lcrV* and *shlAB* loci. LcrV is a translocator of the contact-dependent type III secretion system that is required for full virulence in *Y. pseudotuberculosis* (Bröms et al., 2007). The type III secretion system of *Y. psedotuberculosis* impedes phagocytosis through the insertion of Yop effectors into macrophages (Atkinson and Williams, 2016). The *shlAB* locus is well characterized in *Serratia marcescens*, where it encodes a type V secretion system that promotes autophagy into non-phagocytic eukaryotic cells during intracellular invasion (Di Venanzio et al., 2014). The *ShlAB* encoded system plays a key role in *S. marcescens* virulence (Lin et al., 2010; Di Venanzio et al., 2014); however, its role in *Y. pseudotuberculosis* remains unknown and should be further explored.

*Yersinia pseudotuberculosis* strains have varying capabilities to form biofilms (Darby et al., 2002; Joshua et al., 2003; Erickson et al., 2006; Sun et al., 2008). Our results confirmed that all farm isolates were capable of forming biofilm *in vitro*, although the robustness of the biofilms varied between isolates. All isolates from packaged raw milk formed robust biofilms, whereas isolates from bulk tank milk, and milk filters separated into two distinct biofilm-forming phenotypes. Three of the poorest biofilm-formers lacked the virulence plasmid pYV. Indeed, the pYV virulence gene *yadA* facilitates the adhesion of *Y. pseudotuberculosis* on biotic and abiotic surfaces (Perregaard et al., 1991). Isolates cured of *yadA* or the entire pYV have inferior biofilm forming abilities to isolates with the complete pYV. Several biofilm-associated genes, including *yadA*, contained non-synonymous mutations between ST42 and ST43, which potentially contributed to the better biofilm formation by the ST43 isolates containing pYV in contrast to the ST42 fecal isolates. As the rate of biofilm development may vary between strains, monitoring biofilm formation over a longer incubation period could had provided additional insight into the strain variability. A 24 h incubation period was chosen for this study on the grounds that IP32953 biofilms grown on 96-well plates at 28°C reach peak optical density at approximately 24 h, after which the biofilm no longer increases in mass (Hinchliffe et al., 2008).

Adaptation to detergents and disinfectants may play a role in the survival and persistence of *Y. pseudotuberculosis* in the milking system. The outbreak farm staff cleaned the milking system thrice daily, rotating between alkaline and acidic cleaning solutions. Non-synonymous mutations differentiating the ST43 outbreak cluster from the ST42 fecal isolates were present in genes associated with acid (*clpV*) and alkaline (*nhaB*) stress response. Although phenotypic screening was not performed to confirm this, the genomic diversity could facilitate differential sensitivities toward the biocides used to clean the milking system.

Although all *Y. pseudotuberculosis* appear to grow at low temperatures, significant diversity in growth fitness exists among wild-type strains (Keto-Timonen et al., 2017). In the present study, ST43 outbreak cluster demonstrated significantly better growth fitness at 3°C than the ST42 fecal isolates, suggesting that the outbreak strain was better adapted to survival in a cold (abiotic) environment than the non-outbreak strains. Genotypic characterization of the farm isolates demonstrated that the genes *cheA* and *rstB* contained multiple non-synonymous mutations between the ST42 and ST43 farm isolates. The transcription of these genes increases significantly during cold shock, and the deletion of *cheA* significantly reduced the growth fitness of IP32953 at 3°C (Palonen et al., 2011). Genome-wide association studies could further illuminate which genomic variants contribute to advantageous phenotypes for survival and persistence.

Pärn et al. (2015) investigated the growth of *Y. pseudotuberculosis* at 4°C in bulk tank milk naturally contaminated with the outbreak strain at a level of 2 CFU/ml. The authors used the growth data to infer that the infective dose for a patient who consumed <1 dl of milk ≤3 days after the packaging date was between 10^3^ and 10^4^ CFU. Retail and consumer storage temperatures were not available for the outbreak investigations; however, a Finnish consumer survey suggested that the average storage temperature of raw milk is 6°C (Perkiömäki et al., 2012). In the present study, the outbreak strain grew in packaged raw milk stored at 6°C from a mean initial level of 4 CFU/ml (range 0–10 CFU/ml) to a mean level of 64 CFU/ml (range 3–250 CFU/ml) in 3 days, supporting the infective dose estimate of Pärn et al. (2015). In contrast to the marked biological variability among replicates, little variability was present between raw milk package types, particularly at the higher inoculum levels. However, the large size of the 3-liter bag-in-box may promote longer consumer storage times than the smaller 1-liter bottles, in turn increasing the risk associated with psychrotrophic pathogens (Castro et al., 2017). The rapid growth of *Y. pseudotuberculosis* in refrigerated raw milk implies that raw milk storage should be avoided. Moreover, elimination of *Y. pseudotuberculosis* by heat treatment of raw milk prior to consumption is an effective risk management strategy.

## Conclusion

The present study provided a novel insight into the poorly understood epidemiology and characteristics of *Y. pseudotuberculosis* in dairy production. We demonstrated that the outbreak strain persisted on the source farm and had strain characteristics favorable for persistence, namely the ability to form biofilm *in vitro*, and increased growth fitness at low temperature. Moreover, the outbreak strain grew rapidly in refrigerated packaged milk. Effective pest control in dairy production, improved consumer awareness of raw milk risks, and heat treatment of raw milk prior to consumption could help prevent future outbreaks of *Y. pseudotuberculosis* from unpasteurized drinking milk.

## Author Contributions

HC, ML, and SH contributed to conception and design of the study. HC, AH, and SH performed the experiments. HC, AJ, and JI contributed to whole genome sequencing and genome analysis. MH, HK, ML, and SH provided materials for the study. HC wrote the manuscript. All authors contributed to manuscript revision and approved the submitted version.

## Conflict of Interest Statement

The authors declare that the research was conducted in the absence of any commercial or financial relationships that could be construed as a potential conflict of interest.

## Footnotes

1 http://www.combase.cc/
