# Supplementary material for: Genomic Epidemiology and Phenotyping Reveal on-Farm Persistence and Cold Adaptation of Raw Milk Outbreak-Associated Yersinia pseudotuberculosis
Source: Front Microbiol. 2019 May 14;10:1049. doi: 10.3389/fmicb.2019.01049 (PMC6528616; doi:10.3389/fmicb.2019.01049)
Supplement: Supplementary file 1 [file Table_1.DOCX]

**Table S1**. Assembly statistics and accession numbers of the *Y. pseudotuberculosis* isolates
sequenced in the present study.

| **Strain ID** | **Total length (Mb)** | **Number of contigs** | **GC content (%)** | **N50 (bp)** | **Mean depth of coverage** | **Read accession** |
| --- | --- | --- | --- | --- | --- | --- |
| S1 | 4.6 | 215 | 47.5 | 47507 | 90.8 | ERR2713003 |
| S2 | 4.7 | 209 | 47.6 | 47983 | 108.2 | ERR2713004 |
| S4 | 4.5 | 284 | 47.5 | 32103 | 77.8 | ERR2713005 |
| S7 | 4.5 | 268 | 47.5 | 33951 | 90.5 | ERR2713006 |
| S8 | 4.6 | 188 | 47.4 | 67305 | 106.0 | ERR2713007 |
| S9 | 4.6 | 202 | 47.5 | 57331 | 105.0 | ERR2713008 |
| S10 | 4.6 | 147 | 47.4 | 82815 | 128.2 | ERR2713009 |
| S13 | 4.6 | 167 | 47.4 | 74426 | 89.5 | ERR2713010 |
| S18 | 4.5 | 190 | 47.5 | 42780 | 114.4 | ERR2713011 |
| S23 | 4.5 | 199 | 47.5 | 57153 | 100.0 | ERR2713012 |
| S24 | 4.7 | 240 | 47.6 | 42583 | 78.7 | ERR2713013 |
| S25 | 4.7 | 246 | 47.6 | 37304 | 83.7 | ERR2713014 |
| S26 | 4.7 | 247 | 47.6 | 40135 | 76.3 | ERR2713015 |
| S27 | 4.5 | 165 | 47.4 | 68491 | 117.7 | ERR2713016 |
