# Supplementary material for: Genomic Epidemiology and Phenotyping Reveal on-Farm Persistence and Cold Adaptation of Raw Milk Outbreak-Associated Yersinia pseudotuberculosis
Source: Front Microbiol. 2019 May 14;10:1049. doi: 10.3389/fmicb.2019.01049 (PMC6528616; doi:10.3389/fmicb.2019.01049)
Supplement: Supplementary file 2 [file Table_2.DOCX]

| **Table S2.** *Y. pseudotuberculosis* ST43 isolates selected for phylogenomic analyses (n=38). | | | | | |
| --- | --- | --- | --- | --- | --- |
| **Accession** | **Name** | **Country** | **Year** | **Origin** | **Publication** |
| ERR2713003 | S1 | Finland | 2014 | Bulk tank milk | This study |
| ERR2713004 | S2 | Finland | 2014 | Milk filter | This study |
| ERR2713005 | S4 | Finland | 2014 | Milk package | This study |
| ERR2713006 | S7 | Finland | 2014 | Milk package | This study |
| ERR2713007 | S8 | Finland | 2014 | Bulk tank milk | This study |
| ERR2713008 | S9 | Finland | 2014 | Milk filter | This study |
| ERR2713009 | S10 | Finland | 2014 | Milk package | This study |
| ERR2713010 | S13 | Finland | 2014 | Milk filter | This study |
| ERR2713011 | S18 | Finland | 2014 | Bulk tank milk | This study |
| ERR2713012 | S23 | Finland | 2014 | Milk filter | This study |
| ERR1413979 | NZYP4680 | New Zealand | 2006 | Human | Williamson et al., 2017 |
| ERR1413988 | NZYP4689 | New Zealand | 2015 | Human | Williamson et al., 2017 |
| ERR1414000 | NZYP4701 | New Zealand | 2013 | Human | Williamson et al., 2017 |
| ERR1414001 | NZYP4702 | New Zealand | 2013 | Human | Williamson et al., 2017 |
| ERR1414002 | NZYP4703 | New Zealand | 2013 | Human | Williamson et al., 2017 |
| ERR1414064 | NZYP4766 | New Zealand | 2014 | Human | Williamson et al., 2017 |
| ERR1414093 | NZYP4795 | New Zealand | 2010 | Human | Williamson et al., 2017 |
| ERR1414098 | NZYP8106 | New Zealand | 2006 | Human | Williamson et al., 2017 |
| ERR1447957 | 2812 | Finland | 1979 | Human | Seecharran et al., ‎2017 |
| ERR1447965 | 2887 | Italy |  | Hare | Seecharran et al., ‎2017 |
| ERR1447966 | 2889 | Italy |  | Poultry | Seecharran et al., ‎2017 |
| ERR1447982 | Tytgat | Belgium |  | Human | Seecharran et al., ‎2017 |
| ERR1447983 | H749-36/89 | Germany | 1989 | Duck | Seecharran et al., ‎2017 |
| ERR1448011 | H938-36/89 | Germany | 1989 | Hare | Seecharran et al., ‎2017 |
| ERR1448012 | H305-36/89 | Australia | 1989 | Deer | Seecharran et al., ‎2017 |
| ERR1448016 | Y.PT/7 | Belgium |  | Human | Seecharran et al., ‎2017 |
| ERR1448023 | 2895 | Italy |  | Bird | Seecharran et al., ‎2017 |
| ERR1448024 | 2497 | Italy |  | Hare | Seecharran et al., ‎2017 |
| ERR1448029 | G2/77/2 | Denmark | 1977 | Bird | Seecharran et al., ‎2017 |
| ERR1448030 | G798/82/1 | Denmark | 1982 | Bird | Seecharran et al., ‎2017 |
| ERR1448038 | 866/81 | Finland | 1981 | Human | Seecharran et al., ‎2017 |
| ERR1448039 | 866/81 | Finland | 1981 | Human | Seecharran et al., ‎2017 |
| ERR1448048 | 2812/1998 | Finland | 1998 | Pigeon | Seecharran et al., ‎2017 |
| ERR1448053 | 3858/2000 | Finland | 2000 | Hare | Seecharran et al., ‎2017 |
| ERR1448054 | 3876/2001 | Finland | 2001 | Hare | Seecharran et al., ‎2017 |
| ERR1448063 | IP33290 | France |  | Human | Seecharran et al., ‎2017 |
| ERR1448064 | 42/00 | Sweden | 2000 | Human | Seecharran et al., ‎2017 |
| ERR1448068 | 36/83 | Finland | 1984 | Human | Seecharran et al., ‎2017 |
