# Supplementary material for: Genomic Epidemiology and Phenotyping Reveal on-Farm Persistence and Cold Adaptation of Raw Milk Outbreak-Associated Yersinia pseudotuberculosis
Source: Front Microbiol. 2019 May 14;10:1049. doi: 10.3389/fmicb.2019.01049 (PMC6528616; doi:10.3389/fmicb.2019.01049)
Supplement: Supplementary file 3 [file Table_3.DOCX]

| **Table S3.** *Y. pseudotuberculosis* ST42 strains selected for phylogenomic analyses (n=31). | | | | | | |
| --- | --- | --- | --- | --- | --- | --- |
| **Accession** | **Name** | | **Country** | **Year** | **Origin** | **Publication** |
| ERR2713013 | S24 | | Finland | 2014 | Feces | This study |
| ERR2713014 | S25 | | Finland | 2014 | Feces | This study |
| ERR2713015 | S26 | | Finland | 2014 | Feces | This study |
| ERR2713016 | S27 | | Finland | 2014 | Feces | This study |
| ERR1413976 | NZYP4713 | | New Zealand | 2014 | Human | Williamson et al., 2017 |
| ERR1413980 | NZYP4681 | | New Zealand | 2006 | Human | Williamson et al., 2017 |
| ERR1413994 | NZYP4695 | | New Zealand | 2011 | Human | Williamson et al., 2017 |
| ERR1414100 | NZYP8108 | | New Zealand | 2015 | Human | Williamson et al., 2017 |
| ERR1414101 | NZYP8109 | | New Zealand | 2015 | Human | Williamson et al., 2017 |
| ERR1447954 | 11J | | France |  | Human | Seecharran et al., ‎2017 |
| ERR1447959 | 15193/74 | | Finland | 1974 | Human | Seecharran et al., ‎2017 |
| ERR1447986 | RU488 | | Russia |  | Salmon | Seecharran et al., ‎2017 |
| ERR1447992 | 104 | | Italy |  | Pidgeon | Seecharran et al., ‎2017 |
| ERR1447993 | 103 | | Italy |  | Small mammal | Seecharran et al., ‎2017 |
| ERR1447996 | Rollier | | Belgium |  | Human | Seecharran et al., ‎2017 |
| ERR1447999 | H655-36/87 | | Germany | 1987 | Human | Seecharran et al., ‎2017 |
| ERR1448001 | 488 | | Russia |  | Salmon | Seecharran et al., ‎2017 |
| ERR1448002 | 514 | | Russia |  | Salmon | Seecharran et al., ‎2017 |
| ERR1448004 | Y.PT/8 | | Belgium |  | Human | Seecharran et al., ‎2017 |
| ERR1448008 | 2886 | | Italy |  | Hare | Seecharran et al., ‎2017 |
| ERR1448010 | 504/72 | | Italy | 1972 | Duck | Seecharran et al., ‎2017 |
| ERR1448046 | 2800/1998 | | Finland | 1998 | Jack daw | Seecharran et al., ‎2017 |
| ERR1448047 | 2809/1998 | | Finland | 1998 | Small mammal | Seecharran et al., ‎2017 |
| ERR1448049 | 2814/1998 | | Finland | 1998 | Hare | Seecharran et al., ‎2017 |
| ERR1448052 | 3822/2000 | | Finland | 2000 | Hare | Seecharran et al., ‎2017 |
| ERR1448059 | 2512/2005 | | Finland | 2005 | Wild animal | Seecharran et al., ‎2017 |
| ERR1448065 | 1180/95 | | Sweden | 1995 | Human | Seecharran et al., ‎2017 |
| ERR1448066 | 921/93 | | Sweden | 1993 | Human | Seecharran et al., ‎2017 |
| ERR1448075 | 496/84 | | Finland | 1984 | Human | Seecharran et al., ‎2017 |
| SRR1922795 | YPT-A00003 | | USA |  | Human | Reuter et. al., 2014 |
| NZ_CP009712 | IP32953 | | France |  | Human | Johnson et al., 2015 |
|  | |  |  |  |  |  |
