# Supplementary material for: Genomic Epidemiology and Phenotyping Reveal on-Farm Persistence and Cold Adaptation of Raw Milk Outbreak-Associated Yersinia pseudotuberculosis
Source: Front Microbiol. 2019 May 14;10:1049. doi: 10.3389/fmicb.2019.01049 (PMC6528616; doi:10.3389/fmicb.2019.01049)
Supplement: Supplementary file 4 [file Table_4.DOCX]

**Table S4.** Allelic diversity between the ST43 outbreak-associated *Y. pseudotuberculosis* isolates (n=10), ST42 fecal isolates (n=4), and the reference strain IP32953
 in 110 genes associated with virulence, stress response, biofilm formation and motility.

| **Gene name** | **RefSeq protein ID** | **Refseq  locus tag** | **Functional role** | | | **SNPs ^a^** | | **BLASTx   identity ^b^** | | **Reference** |
| --- | --- | --- | --- | --- | --- | --- | --- | --- | --- | --- |
|  |  |  | **virulence** | **stress response** | **biofilm /motility** | **ST42** | **ST43** | **ST42** | **ST43** |  |
| *adiA* | WP_011191987 | YPTB_RS06865 |  | ✓ |  | 0 | 0 | 100 % | 100 % | Chen et al., 2016 |
| *adiC* | WP_011191986 | YPTB_RS06860 |  | ✓ |  | 0 | 0 | 100 % | 100 % | Chen et al., 2016 |
| *ail* | WP_012104793 | YPTB_RS15505 | ✓ |  |  | 0 | 0 | 100 % | 100 % | Revel & Miller 2001 |
| *arcA* | WP_002209234 | YPTB_RS03390 |  | ✓ |  | 0 | 0 | 100 % | 100 % | Chen et al., 2016 |
| *arcB* | WP_002210142 | YPTB_RS18945 |  | ✓ |  | 0 | 2 | 100 % | 100 % | Chen et al., 2016 |
| *arnA* | WP_011192542 | YPTB_RS12690 |  | ✓ |  | 0 | 0 | 100 % | 100 % | Chen et al., 2016 |
| *arnB* | WP_002211825 | YPTB_RS12700 |  | ✓ |  | 0 | 1 | 100 % | 100 % | Chen et al., 2016 |
| *ascD* | WP_011191878 | YPTB_RS05510 |  |  | ✓ | 0 | 6 | 100 % | 99 % | Joshua et al., 2015 |
| *aspA* | WP_002230464 | YPTB_RS02315 |  | ✓ |  | 0 | 5 | 100 % | 100 % | Chen et al., 2016 |
| *cckA* | WP_011193265 | YPTB_RS20595 |  |  | ✓ | 9 | 0 | 99 % | 100 % | Joshua et al., 2015 |
| *cheA* | WP_041175460 | YPTB_RS13085 | ✓ | ✓ |  | 0 | 8 | 100 % | 99 % | Palonen et al. 2011 |
| *cheY* | WP_041175460 | YPTB_RS13045 | ✓ | ✓ |  | 1 | 0 | 100 % | 100 % | Palonen et al. 2011 |
| *clpV* | WP_002214742 | YPTB_RS03625 | ✓ | ✓ |  | 0 | 6 | 100 % | 99 % | Chen et al., 2016 |
| *clsA* | WP_011192437 | YPTB_RS11560 | ✓ |  |  | 0 | 3 | 100 % | 99 % | Revel & Miller 2001 |
| *cra* | WP_005156888 | YPTB_RS03800 |  | ✓ |  | 0 | 6 | 100 % | 100 % | Chen et al., 2016 |
| *csdA* | WP_002209261 | YPTB_RS02780 |  | ✓ |  | 0 | 0 | 100 % | 100 % | Palonen et al. 2011 |
| *cspA1* | WP_011193172 | YPTB_RS19390 |  | ✓ |  | 0 | 1 | 100 % | 100 % | Keto-Timonen et al. 2016 |
| *cspA2* | WP_011193173 | YPTB_RS19400 |  | ✓ |  | 0 | 0 | 100 % | 100 % | Keto-Timonen et al. 2016 |
| *cspB1* | WP_002212220 | YPTB_RS15955 |  | ✓ |  | 0 | 0 | 100 % | 100 % | Keto-Timonen et al. 2016 |
| *cspB2* | WP_002211317 | YPTB_RS07855 |  | ✓ |  | 0 | 0 | 100 % | 100 % | Keto-Timonen et al. 2016 |
| *cspC1* | WP_002221949 | YPTB_RS08960 |  | ✓ |  | 0 | 1 | 100 % | 100 % | Keto-Timonen et al. 2016 |
| *cspC2* | WP_002210893 | YPTB_RS13135 |  | ✓ |  | 0 | 0 | 100 % | 100 % | Keto-Timonen et al. 2016 |
| *cspD* | WP_002211350 | YPTB_RS07690 |  | ✓ |  | 0 | 0 | 100 % | 100 % | Keto-Timonen et al. 2016 |
| *cspE* | WP_002210315 | YPTB_RS05995 |  | ✓ |  | 0 | 0 | 100 % | 100 % | Keto-Timonen et al. 2016 |
| *csrA* | WP_002209449 | YPTB_RS04570 | ✓ | ✓ |  | 0 | 0 | 100 % | 100 % | Chen et al., 2016 |
| *dbpA* | WP_011192156 | YPTB_RS09100 |  | ✓ |  | 0 | 0 | 100 % | 100 % | Palonen et al. 2011 |
| *dnaJ* | WP_002209249 | YPTB_RS03445 | ✓ |  |  | 0 | 0 | 100 % | 100 % | Revel & Miller 2001 |
| *flhA* | WP_011192160 | YPTB_RS09175 | ✓ | ✓ | ✓ | 0 | 0 | 100 % | 100 % | Joshua et al., 2015 |
| *flhD* | WP_011192574 | YPTB_RS13105 |  |  | ✓ | 0 | 0 | 100 % | 100 % | Chen et al., 2016 |
| *fliA* | WP_011192188 | YPTB_RS09430 |  | ✓ | ✓ | 0 | 1 | 100 % | 100 % | Joshua et al., 2015 |
| *hfq* | WP_002209151 | YPTB_RS02460 | ✓ | ✓ |  | 0 | 0 | 100 % | 100 % | Galindo et al. 2011 |
| *hmsF* | WP_002212043 | YPTB_RS10700 |  |  | ✓ | 0 | 6 | 100 % | 99 % | Zhao et al., 2017 |
| *hmsH* | WP_011192366 | YPTB_RS10695 |  |  | ✓ | 0 | 3 | 100 % | 100 % | Zhao et al., 2017 |
| *hmsP* | WP_041175480 | YPTB_RS20735 |  |  | ✓ | 5 | 8 | 99 % | 99 % | Zhao et al., 2017 |
| *hmsR* | WP_002224456 | YPTB_RS10705 |  |  | ✓ | 0 | 2 | 100 % | 100 % | Zhao et al., 2017 |
| *hmsS* | WP_011192367 | YPTB_RS10710 |  |  | ✓ | 0 | 2 | 100 % | 99 % | Zhao et al., 2017 |
| *hmsT* | WP_011191678 | YPTB_RS03220 |  |  | ✓ | 0 | 0 | 100 % | 100 % | Zhao et al., 2017 |
| *hutC* | WP_011192375 | YPTB_RS10790 |  |  | ✓ | 0 | 0 | 100 % | 100 % | Joshua et al., 2015 |
| *inv* | WP_042592924 | YPTB_RS09190 | ✓ |  |  | 0 | 0 | 100 % | 100 % | Revel & Miller 2001 |
| *irp1* | WP_011192136 | YPTB_RS08780 | ✓ (HPI) |  |  | 2 | 0 | 100 % | 100 % | Revel & Miller 2001 |
| *irp2* | WP_002212775 | YPTB_RS08785 | ✓ (HPI) |  |  | 0 | 1 | 100 % | 100 % | Revel & Miller 2001 |
| *lcrV* | WP_011191383 | YPTB_RS21700 | ✓(pYV)^c^ |  |  | 13 | 0 | 96 % | 100 % | Atkinson & Williams, 2016 |
| *nlpD* | WP_011191779 | YPTB_RS04305 | ✓ |  |  | 0 | 0 | 100 % | 100 % | Revel & Miller 2001 |
| *ntrC* | WP_011191444 | YPTB_RS00205 |  | ✓ |  | 1 | 0 | 100 % | 100 % | Chen et al., 2016 |
| *nhaA* | WP_011191700 | YPTB_RS03450 |  | ✓ |  | 0 | 0 | 100 % | 100 % | Chen et al., 2016 |
| *nhaB* | WP_011192424 | YPTB_RS11345 |  | ✓ |  | 0 | 30 | 100 % | 99 % | Chen et al., 2016 |
| *ompB* | WP_002208913 | YPTB_RS20345 |  | ✓ | ✓ | 0 | 0 | 100 % | 100 % | Chen et al. 2016 |
| *ompR* | WP_002208914 | YPTB_RS20350 |  | ✓ | ✓ | 0 | 0 | 100 % | 100 % | Chen et al. 2016 |
| *phoP* | WP_002230788 | YPTB_RS13240 | ✓ | ✓ | ✓ | 0 | 1 | 100 % | 100 % | Galindo et al. 2011 |
| *phoQ* | WP_002210918 | YPTB_RS13245 | ✓ | ✓ | ✓ | 0 | 2 | 100 % | 100 % | Galindo et al. 2011 |
| *psaA* | WP_002208794 | YPTB_RS07375 | ✓ |  |  | 0 | 0 | 100 % | 100 % | Revel & Miller 2001 |
| *psaC* | WP_002221750 | YPTB_RS07385 | ✓ |  | ✓ | 0 | 0 | 100 % | 100 % | Revel & Miller 2001 |
| *psn* | WP_000784549 | YPTB_RS08760 | ✓ |  |  | 0 | 0 | 100 % | 100 % | Revel & Miller 2001 |
| *pspC* | WP_002210977 | YPTB_RS12360 | ✓ |  | ✓ | 0 | 0 | 100 % | 100 % | Revel & Miller 2001 |
| *pstC* | WP_002215558 | YPTB_RS21385 | ✓ |  |  | 0 | 0 | 100 % | 100 % | Revel & Miller 2001 |
| *rcsA* | WP_002230951 | YPTB_RS13505 |  | ✓ | ✓ | 0 | 1 | 100 % | 100 % | Chen et al. 2016 |
| *rcsB* | WP_002210824 | YPTB_RS06950 |  | ✓ | ✓ | 0 | 0 | 100 % | 100 % | Chen et al. 2016 |
| *rcsC* | WP_002210142 | YPTB_RS18945 |  | ✓ |  | 2 | 0 | 100 % | 100 % | Chen et al., 2016 |
| *rcsD* | WP_011191996 | YPTB_RS06955 |  | ✓ |  | 0 | 0 | 100 % | 100 % | Chen et al., 2016 |
| *relA* | WP_002209373 | YPTB_RS04180 |  | ✓ | ✓ | 0 | 9 | 100 % | 100 % | Chen et al., 2016 |
| *rhlB* | WP_002228177 | YPTB_RS01005 |  | ✓ |  | 0 | 1 | 100 % | 100 % | Palonen et al. 2011 |
| *rhlE* | WP_002220152 | YPTB_RS06725 |  | ✓ |  | 1 | 0 | 100 % | 100 % | Palonen et al. 2011 |
| *rovA* | WP_002210955 | YPTB_RS12465 | ✓ |  |  | 0 | 0 | 100 % | 100 % | Revel & Miller 2001 |
| *rovM* | WP_002210280 | YPTB_RS14040 | ✓ | ✓ | ✓ | 0 | 1 | 100 % | 100 % | Chen et al., 2016 |
| *rpoE* | WP_002209672 | YPTB_RS15655 |  | ✓ |  | 0 | 0 | 100 % | 100 % | Palonen et al. 2011 |
| *rseA* | WP_002209673 | YPTB_RS15650 |  | ✓ |  | 0 | 0 | 100 % | 100 % | Palonen et al. 2011 |
| *rseB* | WP_002209674 | YPTB_RS15645 |  | ✓ |  | 0 | 0 | 100 % | 100 % | Palonen et al. 2011 |
| *rsmA* | WP_011191710 | YPTB_RS03555 | ✓ |  |  | 0 | 1 | 100 % | 100 % | Revel & Miller 2001 |
| *rstA* | WP_002211015 | YPTB_RS12165 |  | ✓ |  | 0 | 2 | 100 % | 100 % | Chen et al., 2016 |
| *rstB* | WP_011192505 | YPTB_RS12170 |  | ✓ |  | 5 | 0 | 99 % | 100 % | Chen et al., 2016 |
| *shlA1* | WP_011192407 | YPTB_RS11125 | ✓(?) |  |  | 15 | 0 | 99 % | 100 % | Di Venanzio et al., 2014 |
| *shlA2* | WP_011193208 | YPTB_RS19745 | ✓(?) |  |  | 28 | 0 | 99 % | 100 % | Di Venanzio et al., 2014 |
| *shlB1* | WP_011192406 | YPTB_RS11120 | ✓(?)^d^ |  |  | 128 | 0 | 91 % | 100 % | Di Venanzio et al., 2014 |
| *shlB2* | WP_002230618 | YPTB_RS19740 | ✓(?) |  |  | 8 | 1 | 99 % | 99 % | Di Venanzio et al., 2014 |
| *sodA* | WP_002209614 | YPTB_RS21190 | ✓ |  |  | 0 | 0 | 100 % | 100 % | Revel & Miller 2001 |
| *spoT* | WP_002209002 | YPTB_RS00275 |  | ✓ | ✓ | 0 | 0 | 100 % | 100 % | Chen et al., 2016 |
| *srmB* | WP_002209669 | YPTB_RS15670 |  | ✓ |  | 0 | 0 | 100 % | 100 % | Palonen et al. 2011 |
| *tatC* | WP_011191543 | YPTB_RS01515 |  | ✓ | ✓ | 0 | 0 | 100 % | 100 % | Chen et al., 2016 |
| *topA* | WP_011192446 | YPTB_RS11700 | ✓ |  |  | 0 | 1 | 100 % | 99 % | Revel & Miller 2001 |
| *ureA* | WP_002215288 | YPTB_RS15925 |  | ✓ |  | 0 | 0 | 100 % | 100 % | Chen et al., 2016 |
| *ureB* | WP_002212228 | YPTB_RS15920 |  | ✓ |  | 0 | 0 | 100 % | 100 % | Chen et al., 2016 |
| *ureC* | WP_002212229 | YPTB_RS15915 |  | ✓ |  | 0 | 0 | 100 % | 100 % | Chen et al., 2016 |
| *yadA* | WP_011191362 | YPTB_RS21525 | ✓(pYV) |  | ✓ | 0 | 3 | 100 % | 99 % | Atkinson & Williams, 2016 |
| *ybtA* | WP_000140406 | YPTB_RS08790 | ✓ (HPI) |  |  | 0 | 1 | 100 % | 100 % | Revel & Miller 2001 |
| *ybtE* | WP_001088826 | YPTB_RS08765 | ✓ (HPI) |  |  | 0 | 0 | 100 % | 100 % | Revel & Miller 2001 |
| *ybtP* | WP_012413641 | YPTB_RS08795 | ✓ (HPI)^e^ |  |  | 0 | 1 | 100 % | 99 % | Revel & Miller 2001 |
| *ybtQ* | WP_002212761 | YPTB_RS08800 | ✓ (HPI) |  |  | 0 | 0 | 100 % | 100 % | Revel & Miller 2001 |
| *ybtS* | WP_000703040 | YPTB_RS08810 | ✓ (HPI) |  |  | 0 | 0 | 100 % | 100 % | Revel & Miller 2001 |
| *ybtT* | WP_002212799 | YPTB_RS08770 | ✓ (HPI) |  |  | 0 | 0 | 100 % | 100 % | Revel & Miller 2001 |
| *ybtU* | WP_000982866 | YPTB_RS08775 | ✓ (HPI) |  |  | 0 | 0 | 100 % | 100 % | Revel & Miller 2001 |
| *ybtX* | WP_001286280 | YPTB_RS08805 | ✓ (HPI) |  |  | 0 | 0 | 100 % | 100 % | Revel & Miller 2001 |
| *yfhA* | WP_002211880 | YPTB_RS08500 |  | ✓ |  | 0 | 0 | 100 % | 100 % | Chen et al., 2016 |
| *ymoA* | WP_002208622 | YPTB_RS05410 | ✓ |  |  | 0 | 0 | 100 % | 100 % | Revel & Miller 2001 |
| *yopB* | WP_011191382 | YPTB_RS21690 | ✓(pYV) |  |  | 2 | 0 | 100 % | 100 % | Atkinson & Williams, 2016 |
| *yopD* | WP_002212987 | YPTB_RS21685 | ✓(pYV) |  |  | 0 | 0 | 100 % | 100 % | Atkinson & Williams, 2016 |
| *yopE* | WP_002353311 | YPTB_RS21595 | ✓(pYV) |  |  | 0 | 0 | 100 % | 100 % | Atkinson & Williams, 2016 |
| *yopH* | WP_011191390 | YPTB_RS21830 | ✓(pYV) |  |  | 0 | 0 | 100 % | 100 % | Atkinson & Williams, 2016 |
| *yopJ* | WP_002212912 | YPTB_RS21840 | ✓(pYV) |  |  | 0 | 0 | 100 % | 100 % | Atkinson & Williams, 2016 |
| *yopM* | WP_002212907 | YPTB_RS21855 | ✓(pYV) |  |  | 0 | 0 | 100 % | 100 % | Atkinson & Williams, 2016 |
| *yopT* | WP_002212938 | YPTB_RS21775 | ✓(pYV) |  |  | 0 | 0 | 100 % | 100 % | Atkinson & Williams, 2016 |
| *ypkA* | WP_011191359 | YPTB_RS21465 | ✓(pYV) |  |  | 0 | 3 | 100 % | 100 % | Atkinson & Williams, 2016 |
| *ypsI* | WP_002211858 | YPTB_RS13575 |  | ✓ | ✓ | 0 | 0 | 100 % | 100 % | Joshua et al., 2015 |
| *ypsR* | WP_002211859 | YPTB_RS13580 |  |  | ✓ | 0 | 0 | 100 % | 100 % | Joshua et al., 2015 |
| *yscF* | WP_002212916 | YPTB_RS21820 | ✓(pYV) |  |  | 0 | 0 | 100 % | 100 % | Atkinson & Williams, 2016 |
| *yscN* | WP_002212955 | YPTB_RS21745 | ✓(pYV) |  |  | 0 | 0 | 100 % | 100 % | Atkinson & Williams, 2016 |
| *yscP* | WP_011191386 | YPTB_RS21755 | ✓(pYV) |  |  | 0 | 1 | 100 % | 99 % | Atkinson & Williams, 2016 |
| *yscQ* | WP_002212948 | YPTB_RS21760 | ✓(pYV) |  |  | 0 | 3 | 100 % | 99 % | Atkinson & Williams, 2016 |
| *ytbI* | WP_002215903 | YPTB_RS17640 |  |  | ✓ | 0 | 0 | 100 % | 100 % | Joshua et al., 2015 |
| *ytbR* | WP_002211655 | YPTB_RS17645 |  | ✓ | ✓ | 0 | 0 | 100 % | 100 % | Joshua et al., 2015 |
| *zntR* | WP_002215702 | YPTB_RS19880 | ✓ | ✓ |  | 0 | 0 | 100 % | 100 % | Wang et al., 2017 |

^a^SNPs: Number of single nucleotide polymorphisms between the Refseq sequence and the ST42/ST32 farm isolates ^b^BLASTx identity: Identity (%) between translated nucleotide sequences of the ST42/ST43 farm isolates and the Refseq protein sequences, derived using NCBI BLASTx
 ^c^(pYV): virulence gene associated with plasmid pYV
^d^(?): virulence gene characterized in *Serratia*; role in *Y. pseudotuberculosis* unknown
^e^ (HPI): virulence gene associated with the *Y. pseudotuberculosis* high pathogenicity island
