# Supplementary material for: Genomic Epidemiology and Phenotyping Reveal on-Farm Persistence and Cold Adaptation of Raw Milk Outbreak-Associated Yersinia pseudotuberculosis
Source: Front Microbiol. 2019 May 14;10:1049. doi: 10.3389/fmicb.2019.01049 (PMC6528616; doi:10.3389/fmicb.2019.01049)
Supplement: Supplementary file 5 [file Table_5.DOCX]

**Table S5.** Farm isolates selected for the growth study at 3 °C.

| ID | **Source** | **Sampling date** | **PFGE^a^** |
| --- | --- | --- | --- |
| S1 | Bulk tank milk | 7.4.2014 | S-1/N-1 |
| S2 | Milk filter | 7.4.2014 | S-1/N-1 |
| S3 | Milk filter | 7.4.2014 | S-1/N-1 |
| S4 | Milk package | 7.4.2014 | S-1/N-1 |
| S5 | Milk package | 7.4.2014 | S-1/N-1 |
| S6 | Milk package | 7.4.2014 | S-1/N-1 |
| S7 | Milk package | 7.4.2014 | S-1/N-1 |
| S8 | Bulk tank milk | 23.4.2014 | S-1/N-1 |
| S9 | Milk filter | 23.4.2014 | S-1/N-1 |
| S10 | Milk package | 29.4.2014 | S-1/N-1 |
| S11 | Milk package | 29.4.2014 | S-1/N-1 |
| S12 | Milk package | 29.4.2014 | S-1/N-1 |
| S13 | Milk filter | 26.5.2014 | S-1/N-1 |
| S14 | Milk filter | 26.5.2014 | S-1/N-1 |
| S15 | Milk filter | 26.5.2014 | S-1/N-1 |
| S16 | Milk filter | 9.10.2014 | S-1/N-1 |
| S17 | Milk filter | 9.10.2014 | S-1/N-1 |
| S18 | Bulk tank milk | 21.10.2014 | S-1/N-1 |
| S19 | Bulk tank milk | 21.10.2014 | S-1/N-1 |
| S20 | Milk filter | 21.10.2014 | S-1/N-1 |
| S21 | Milk filter | 21.10.2014 | S-1/N-1 |
| S22 | Milk filter | 17.11.2014 | S-1/N-1 |
| S23 | Milk filter | 17.11.2014 | S-1/N-1 |
| S24 | Cow feces | 7.4.2014 | S-2/N-2 |
| S25 | Cow feces | 7.4.2014 | S-2/N-2 |
| S26 | Cow feces | 7.4.2014 | S-2/N-2 |
| S27 | Cow feces | 16.6.2014 | S-3/N-3 |
| S28 | Cow feces | 16.6.2014 | S-3/N-3 |
| S29 | Cow feces | 16.6.2014 | S-3/N-3 |

^a^PFGE: pulsed-field gel electrophoresis
