# Supplementary figures and images for: Genomic Epidemiology and Phenotyping Reveal on-Farm Persistence and Cold Adaptation of Raw Milk Outbreak-Associated Yersinia pseudotuberculosis
Source: Front Microbiol. 2019 May 14;10:1049. doi: 10.3389/fmicb.2019.01049 (PMC6528616; doi:10.3389/fmicb.2019.01049)

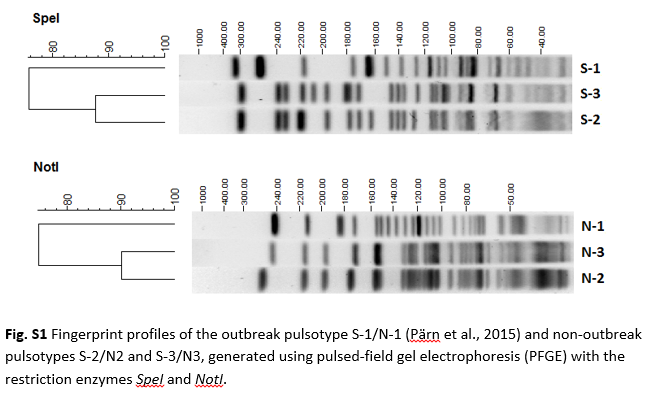

Supplement: Supplementary file 6 [file Image_1.PNG]

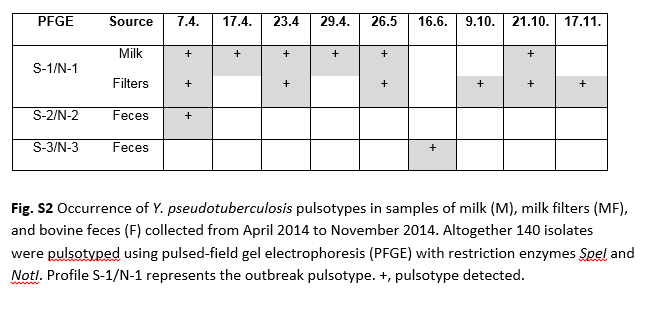

Supplement: Supplementary file 7 [file Image_2.PNG]
